# Individual and Area-Based Socioeconomic Factors Associated With Dementia Incidence in England: Evidence From a 12-Year Follow-up in the English Longitudinal Study of Ageing

**DOI:** 10.1001/jamapsychiatry.2018.1012

**Published:** 2018-05-16

**Authors:** Dorina Cadar, Camille Lassale, Hilary Davies, David J. Llewellyn, G. David Batty, Andrew Steptoe

**Affiliations:** 1 Department of Behavioural Science and Health, University College London, London, United Kingdom; 2 Department of Epidemiology and Public Health, University College London, London, United Kingdom; 3 School of Health Sciences, University of Surrey, Guildford, United Kingdom; 4 Medical School, University of Exeter, Devon, South West England, United Kingdom

## Abstract

**Importance:**

Lower educational attainment is associated with a higher risk of dementia. However, less clear is the extent to which other socioeconomic markers contribute to dementia risk.

**Objective:**

To examine the relationship of education, wealth, and area-based deprivation with the incidence of dementia over the last decade in England and investigate differences between people born in different periods.

**Design, Setting, and Participants:**

Data from the English Longitudinal Study of Ageing, a prospective cohort study that is representative of the English population, were used to investigate the associations between markers of socioeconomic status (wealth quintiles and the index of multiple deprivation) and dementia incidence. To investigate outcomes associated with age cohorts, 2 independent groups were derived using a median split (born between 1902-1925 and 1926-1943).

**Main Outcomes and Measures:**

Dementia as determined by physician diagnosis and the Informant Questionnaire on Cognitive Decline in the Elderly.

**Results:**

A total of 6220 individuals aged 65 years and older enrolled in the study (median [interquartile range] age at baseline, 73.2 [68.1-78.3] years; 3410 [54.8%] female). Of these, 463 individuals (7.4%) had new cases of dementia ascertained in the 12 years between 2002-2003 and 2014-2015. In the cohort born between 1926 and 1943, the hazard of developing dementia was 1.68 times higher (hazard ratio [HR] = 1.68 [95% CI, 1.05-2.86]) for those in the lowest wealth quintile compared with those in the highest quintile, independent of education, index of multiple deprivation, and health indicators. Higher hazards were also observed for those in the second-highest quintile of index of multiple deprivation (HR = 1.62 [95% CI, 1.06-2.46]) compared with those in the lowest (least deprived) quintile.

**Conclusions and Relevance:**

In an English nationally representative sample, the incidence of dementia appeared to be socioeconomically patterned primarily by the level of wealth. This association was somewhat stronger for participants born in later years.

## Key Points

**Question:**

What is the association between various socioeconomic markers and dementia incidence?

**Findings:**

This longitudinal cohort study found that lower wealth in late life, but not education, was associated with increased risk for dementia, suggesting that people with fewer financial resources were at higher risk. No substantive differences were identified in relation to the area of neighborhood deprivation; an age-cohort effect was observed, highlighting that socioeconomic inequalities were more robust among people born in later years.

**Meaning:**

The association between socioeconomic status and dementia incidence in a contemporary cohort of older adults may be driven by wealth rather than education.

## Teaser

This cohort study using data from the English Longitudinal Study of Ageing examines the association of education, wealth, and area-based deprivation with dementia incidence and investigates differences between people born in different periods.

## Introduction

Dementia is one of the most feared medical conditions worldwide; it represents a significant global challenge to health and social care.^1,2^ Recent evidence suggests that dementia rates have decreased in the last few decades in the United Kingdom and other parts of Western Europe.^3,4,5^ Similarly, in the United States, the Framingham Heart Study has shown that age-specific incidence rates of dementia have decreased by almost 20% within the last few decades, and the greatest declines were apparent in individuals with higher educational attainment relative to more basic educational attainment.^6^

Education may serve different roles in the development of dementia: it is a proxy for early-life experiences and (parental) socioeconomic status (SES); it is related to future employment prospects, income, and wealth; it determines occupational exposures and characteristics of adult life (eg, job complexity, work stress, environmental exposures); and it provides lifelong skills for optimal mental abilities and mastery. Education is also thought to be a marker of cognitive reserve, which appears to be protective against cognitive impairment and dementia risk, offering an increased neural network and compensatory mechanisms throughout the life course, even when individuals are facing neuronal death.^7^ Recent systematic reviews have highlighted that low educational level was associated with a higher risk of dementia incidence^8^ as well as with greater risk of dementia-related death.^9^ Some of this evidence highlights that the role of education varies according to period and sociocultural context. The variation in country-specific regulations on compulsory schooling and variations in measurement could account for the differences reported in the literature.

Moreover, given that education is typically completed many decades before dementia onset, other individual and area-based components of SES, such as wealth, income, and area deprivation, may provide a more accurate indication of current socioeconomic resources. Also, at older ages, accumulated wealth represents a more robust measure of socioeconomic resources than income or occupation alone.^10,11^ There are relatively few studies to date that have used socioeconomic indicators other than education to investigate dementia risk. A recent analysis of the Health and Retirement Study compared various SES markers, including parental education (an early-life indicator) and education and income (adult and late-life indicators) associated with late-life memory performance and decline. These findings indicated that income was most strongly associated with decline, although education was the most influential determinant of baseline memory.^12^

Another aspect of socioeconomic position involves neighborhood characteristics and the area of deprivation level, which combines information from multiple domains such as income, employment, education, skills, training, health, disability, crime, and barriers to housing into a single measure. Previous results from the English Longitudinal Study of Ageing (ELSA) showed that the index of multiple deprivation (IMD), the official measure of deprivation in England, was associated with cognitive performance in older age independently of education and SES. These findings indicated that older women had lower cognitive scores if they lived in an area classified in the bottom 20% of IMD when compared with those in the top (least deprived) quintile.^13^ In contrast, Meyer et al^14^ showed that neighborhood SES had limited effects on executive function, independent of personal characteristics such as education and ethnicity. They also showed that individuals with dementia living in neighborhoods with higher SES experienced faster rates of decline before further statistical adjustment for education and ethnicity.^14^ These findings are consistent with the cognitive reserve hypothesis, which acknowledges a rapid cognitive deterioration for people with higher education once the pathological process associated with dementia has been initiated.^7^ However, findings from the Seoul Dementia Management Project^15^ showed there were no additive or synergistic effects between individual-level and district-level of SES, highlighting that the individual level contributed more to the development of cognitive impairment than the district-level SES.

We aimed to describe dementia incidence in a nationally representative cohort of British older adults and to investigate the association with different socioeconomic markers, both via the individual characteristics (education and wealth) and group-level characteristics (IMD). A second objective was to examine the role of socioeconomic markers between 2 independent age cohorts (those born from 1902 to 1925 and from 1926 to 1943).

## Methods

### Data

**Figure 1.**
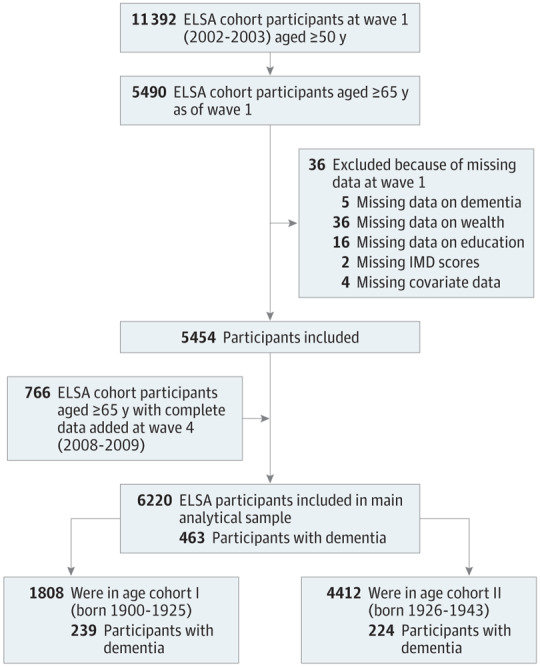
Flowchart of the Individuals Included in Analyses Numbers of excluded persons are nonmutually exclusive. ELSA indicates the English Longitudinal Study of Ageing; IMD, index of multiple deprivations.

The English Longitudinal Study of Ageing (ELSA) is a large, multidisciplinary study representative of the English population both in terms of socioeconomic profile and geographic region.^16^ There have been 7 waves of data collection over a follow-up period of up to 12 years, providing detailed information on health, well-being, and socioeconomic circumstances. We used all the available data spanning 12 years across wave 1 (2002-2003) to wave 7 (2014-2015). Refreshment samples were recruited at waves 3, 4, 6, and 7. For the current analyses, we included only participants aged 65 years and older who were free of dementia at their baseline assessment at either wave 1 or through the refreshment sample of wave 4 (Figure 1 for sample selection).

Ethical approval for each one of the ELSA waves was granted by the National Research Ethics Service (London Multicentre Research Ethics Committee). All participants provided informed consent.

### Study Variables

#### Dementia Ascertainment

Dementia occurrence was determined at each wave using an algorithm based on a combination of self-reported or informant-reported physician diagnosis of dementia or Alzheimer disease or a score above the threshold of 3.38 on the 16**-**question Informant Questionnaire on Cognitive Decline in the Elderly.^17^ This questionnaire is administered to an informant (eg, a family member or a caregiver), who can evaluate the changes in the everyday cognitive function. Each item is scored from 1 (much improved) to 5 (much worse). The validity of this scale was previously examined,^18^ and the threshold used has both high specificity (0.84) and sensitivity (0.82).^19^

#### Socioeconomic Indicators

We measured SES at baseline, including individual characteristics (education and wealth) and area-based characteristics (IMD). Educational attainment was classified into 4 categories: (1) having a university degree or higher; (2) having completed A-levels or the equivalent, which is comparable with high school graduation; (3) having completed education below the A-level; and (4) lacking formal qualifications. Wealth was calculated by summing wealth from property, possessions, housing, investments, savings, artwork, and jewelry, and net of debt^16^; this was divided into quintiles. The index of multiple deprivation (IMD) is a composite measure which combines multiple area-level SES indicators into a single deprivation score.^20^ We used the 2004 IMD for England (in which 1 was least deprived and 5 was most deprived). The highest levels of wealth, education, and IMD were used as the reference group.

#### Covariates

Based on previous findings,^21^ we considered baseline age, sex, marital status (married vs unmarried or widowed), and baseline health (eg, history of stroke, coronary heart disease, hypertension, and diabetes mellitus as potential confounders). Being male, married, and having no health conditions were used as the reference groups.

#### Age Cohorts

To investigate the change in incidence rates over the last decade, we derived 2 groups: age cohort I (who were born between 1902-1925) and age cohort II (who were born between 1926-1943). This derivation was generated using a median split of all birth years (Figure 1).

### Statistical Analyses

Incidence rates of dementia were computed by age and sex per 1000 person-years. We performed χ^2^ tests to ascertain if there were significant differences between SES groups. To summarize the relationship between SES characteristics and dementia incidence, Cox proportional hazards models with age as the underlying time variable were used to calculate hazard ratios (HRs) and accompanying 95% CIs.^22^ We present the results from 4 models: model 1 included unadjusted HRs; model 2 included sex and marital status; model 3 included model 2 with further adjustment for baseline health indicators (stroke, hypertension, diabetes, and cardiovascular disease), and model 4 included model 3 and further adjusted for the additional socioeconomic indicators. We used a forward stepwise approach and the Akaike Information Criterion to select the model of best fit. Given that the original IMD quintile classification was slightly underpowered, we conducted a sensitivity analysis with the IMD regrouped into a binary variable (with quintile 1 [Q1] set to 1 and Q2-Q5 set to 2).

The survival time was calculated using participants’ baseline age at study entry until the age they were found to be experiencing dementia, the point of their death, or the end of the study period (the last wave before dropout, or wave 7, which ran in 2014-2015). The Schoenfeld residual test was used to test the proportional hazards assumption of the models.^23^ For individuals who did not report an exact diagnosis date or for those whose dementia was ascertained with Informant Questionnaire on Cognitive Decline in the Elderly, we considered the midpoint between the wave where dementia was first ascertained and the previous wave where it was not. Mortality data were used for participants who had provided written consent for linkage to official records from the National Health Service central register; the records available the time of these analyses continued until February 2013. All analyses were weighted using the baseline cross-sectional weights derived in ELSA to ensure the sample is representative of the English population.^24^

Given that death is often considered a competing risk for dementia incidence, we conducted supplementary analyses using a modification of the Fine and Gray Subdistribution Hazards model^25^ to account for the competing risk of death, as described elsewhere^26^ (eFigure 1 in the Supplement). All analyses were conducted in Stata SE, Version 14 (StataCorp). Statistical significance was considered to be at or below the .05 level. Additional details are noted in the eAppendix in the Supplement.

## Results

The sample included in these analyses was composed of 6220 individuals, accounting for 43 218 person-years (median follow-up duration, 7 years; range, 1-12 years). Of these, 463 (7.4%) were classified with dementia during the surveillance period, and 1971 (31.7%) died. The baseline median age was 73.2 years (interquartile range, 68.1-78.3 years), while the median age at the time of dementia ascertainment was 82.7 (interquartile range, 78.2-87.8 years). The sample included 6220 people, of whom 3410 (54.8%) were female and 2810 (45.8%) male, 3682 (59.2%) married, and 3288 (52.5%) without formal educational qualifications. Only 1049 of 6220 participants (16.9%) attended university. More men were educated to university degree level than women, while more women had no formal educational qualifications (χ^2^_3_, 338.28; *P* ≤ .001). The baseline median wealth for the overall sample was £15 100 (approximately $21,470; interquartile range [IQR], £2700-£62 546 [$3839-$88 935.30]); for the lowest quintile, the median wealth as £120 (approximately $170.63; IQR, £0-£700 [$0-$995.34]), increasing to £180 000 ($255 936.94; IQR, £117 000-£309 100 [$166 375.78-$439 544.91]) in the highest quintile. Except for stroke, which showed no clear SES gradient, all other health conditions (cardiovascular disease, diabetes, and hypertension) were inversely associated with each one of the SES markers (results presented in eTable 1 of the Supplement).

**Figure 2.**
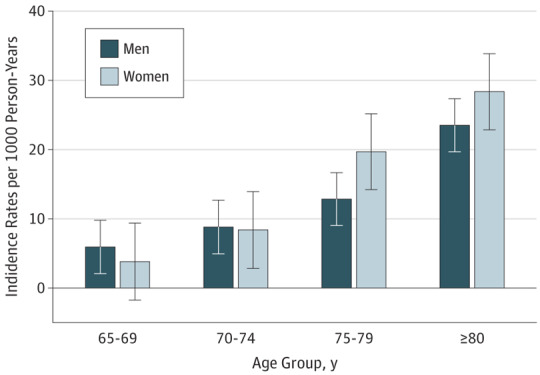
Dementia Incidence Rates Per 1000 Person-Years in Men and Women Presented by Age-Groups in the English Longitudinal Study of Ageing Error bars indicate 95% CIs.

Age-adjusted and sex-adjusted incidence rates for the full ELSA sample and each specific age cohort are presented in Table 1 and Figure 2. The overall incidence rate (IR) was 11.32 per 1000 person-years (95% CI, 10.34-12.41 per 1000 person-years). As anticipated, there was a significant increase in dementia IRs with age from an incidence of 4.38 (95% CI, 3.49-5.57) in people aged 65 to 69 years to 24.69 (95% CI, 21.20-28.91) for those 80 years or older. The comparison between the 2 distinct age-periods cohorts shows a 30% reduction in the IRs of dementia for the overlapping age group of 75 to 79 years who were born between 1902 and 1925 (IR, 20.29; 95% CI, 16.45-25.28) and those born later between 1926 and 1943 (IR, 13.59; 95% CI, 10.33-18.20) (Table 1). There were no significant sex differences in the IRs of dementia (eTable 1 in the Supplement).

**Table 1.** Dementia Incidence Rates Per 1000 Person-Years by Age Cohort

Characteristic	Total Cohort (n = 6220)		Age Cohort I (n = 1808)		Age Cohort II (n = 4412)	
	No. (Cases of Dementia/ Censored)	Incidence Rate (95% CI)	No. (Cases of Dementia/ Censored)	Incidence Rate (95% CI)	No. (Cases of Dementia/ Censored)	Incidence Rate (95% CI)
Total	463/5757	11.32 (10.34-12.41)	239/1569	22.99 (20.31-26.11)	224/4188	7.06 (6.29-8.07)
Age group,y						
65-69	71/2008	4.38 (3.49-5.57)	NA	NA	71/2208	4.38 (3.49-5.57)
70-74	105/1705	8.30 (6.88-10.08)	NA	NA	105/1705	8.30 (6.88-10.09)
75-79	127/963	17.14 (14.49-20.41)	79/518	20.29 (16.45-25.28)	48/475	13.59 (10.33-18.20)
≥80	160/1051	24.69 (21.20-28.91)	160/1501	24.69 (21.20-28.91)	NA	NA
Sex						
Male	187/2623	10.27 (8.92-11.89)	82/655	20.53 (16.64-25.60)	105/1968	7.24 (6.01-8.81)
Female	276/3134	12.09 (10.76-13.63)	157/914	24.39 (20.95-28.55)	119/2220	6.92 (5.80-8.32)
Marital status						
Married	254/3428	9.94 (8.81-11.26)	96/640	21.77 (17.93-26.68)	158/2788	7.35 (6.31-8.61)
Single/divorced	209/2329	13.35 (11.68-15.33)	143/929	23.80 (20.36-28.07)	66/1400	6.48 (5.12-8.34)
Education						
Higher education	73/976	9.85 (7.86-12.50)	37/178	26.22 (19.22-36.58)	36/798	5.72 (4.17-8.08)
A-level	103/1444	9.17 (7.60-11.18)	48/325	19.78 (15.14-26.34)	55/1119	6.06 (4.69-7.99)
>A-level	20/316	9.71 (6.31-15.70)	10/82	23.11 (12.56-46.79)	10/234	5.70 (3.14-11.46)
No qualification	267/3021	13.08 (11.62-14.77)	144/984	23.46 (20.02-27.67)	123/2037	8.32 (7.00-9.97)
Wealth^a^						
Q1 (Highest)	67/1062	7.92 (6.26-10.16)	33/213	19.28 (13.87-27.51)	34/848	4.88 (3.52-6.98)
Q2	82/1096	10.11 (8.16-12.67)	36/230	22.16 (16.18-31.11)	46/866	6.61 (4.99-8.95)
Q3	91/1154	11.03 (9.02-13.64)	49/289	23.33 (17.84-31.03)	42/865	6.66 (4.95-9.16)
Q4	102/1139	12.54 (10.38-15.28)	44/322	21.48 (16.12-29.19)	58/817	9.19 (7.18-11.95)
Q5 (Lowest)	121/1306	15.05 (12.62-18.10)	77/515	26.07 (21.02-32.70)	44/791	8.34 (6.27-11.35)
Index of multiple deprivation^b^						
Q1 (Least deprived)	86/1291	8.62 (7.48-11.24)	49/333	19.27 (14.76-25.59)	37/958	4.82 (3.52-6.77)
Q2	116/1221	12.47 (9.50-14.06)	57/325	25.38 (19.71-33.1)8	59/896	7.92 (6.18-10.30)
Q3	97/1224	11.56 (10.20-15.10)	54/337	25.59 (19.73-33.71)	43/887	6.42 (4.80-8.76)
Q4	90/1109	11.99 (9.21-13.88)	43/314	21.51 (16.20-29.14)	47/795	8.35 (6.34-11.23)
Q5 (Most deprived)	74/913	12.64 (10.70-16.86)	36/260	23.30 (19.97-32.79)	38/652	8.70 (6.37-12.19)
Stroke						
No	407/5170	10.87 (9.87-11.99)	213/1426	22.41 (19.66-25.65)	194/3744	6.68 (5.82-7.71)
Yes	56/587	16.47 (12.78-21.56)	26/143	29.47 (20.40-43.92)	30/444	11.53 (8.16-16.82)
Hypertension						
No	240/3239	11.27 (9.93-12.85)	137/854	24.68 (20.97-29.23)	103/2385	5.64 (4.66-6.88)
Yes	223/2518	12.44 (10.86-14.27)	102/715	20.16 (17.40-25.55)	121/1803	8.95 (7.53-10.73)
Diabetes						
No	416/5215	11.12 (10.11-12.26)	223/1423	23.32 (20.51-26.60)	193/3792	6.64 (5.78-7.67)
Yes	47/542	13.52 (10.28-18.13)	16/146	19.06 (11.95-32.22)	31/396	11.66 (8.34-16.80)
Cardiovascular disease						
No	373/5081	10.17 (9.15-11.34)	193/1360	22.07 (19.24-25.43)	180/3721	6.65 (5.61-7.52)
Yes	90/676	16.21 (13.67-19.36)	46/209	28.00 (21.11-37.82)	44/467	11.03 (8.28-15.01)

Abbreviations: NA, not available; Q, quintile.

^a^ In wealth rankings, Q1 indicates highest wealth category; Q2, the second highest; Q3, the third highest; Q4, the fourth highest; and Q5, the lowest.

^b^ In the index of multiple deprivation, Q1 indicates least deprived; Q2, the second least deprived; Q3, the third least deprived; Q4, the fourth least deprived; and Q5, most deprived.

### Individual and Area-Based Socioeconomic Markers

**Figure 3.**
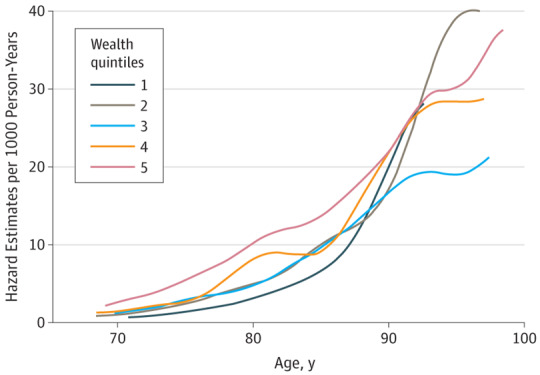
Smoothed Hazard Estimates by Age per 1000 Person-Years by Wealth Quintiles in the English Longitudinal Study of Ageing Wealth quintile 1 indicates the highest level of wealth; quintile 5, the lowest.

The multivariable analyses are summarized in Table 2. Education was not significantly associated with dementia incidence, but wealth was a strong indicator. Per model 4, the hazards of developing dementia were higher for those in the lowest 2 quintiles of wealth (Q4: HR, 1.39; 95% CI, 1.00-1.95; and Q5: HR,  1.50; 95% CI, 1.05-2.13; *P* for trend = .04), compared with those in the highest quintile (Q1), independently of covariates, education, and area-level socioeconomic characteristics (Table 2 and Figure 3).

**Table 2.** Hazard Ratios From Univariate and Multivariate Cox Regression Models by Age Cohort

Characteristic	Hazard Ratios (95% CI) per Model									
	No. (Cases/Censored)	Person-Years	Model 1^**a**^	*P* Value for trend	Model 2^b^	*P* Value for trend	Model 3^c^	*P* Value for trend	Model 4^d^	*P* Value for trend
**ELSA Overall (N = 6220; 43 219 person-years)**										
Education										
University degree	73/976	7974	1 [Reference]	.27	1 [Reference]	.23	1 [Reference]	.32	1 [Reference]	.99
A-level	103/1444	11 593	0.89 (0.67-1.21)		0.90 (0.66-1.23)		0.91 (0.67-1.23)		0.84 (0.62-1.14)	
<A-level	20/316	2270	0.87 (0.53-1.42)		0.86 (0.53-1.41)		0.83 (0.50-1.36)		0.74 (0.44-1.22)	
No qualification	267/3021	21 382	1.07 (0.82-1.39)		1.09 (0.83-1.43)		1.07 (0.81-1.40)		0.92 (0.70-1.23)	
Wealth^e^										
Q1 (Highest)	67/1062	8807	1 [Reference]	.01	1 [Reference]	.002	1 [Reference]	.01	1 [Reference]	.04
Q2	82/1096	8605	1.27 (0.92-1.76)		1.31 (0.94-1.80)		1.29 (0.94-1.79)		1.31 (0.94-1.82)	
Q3	91/1154	8670	1.29 (0.94-1.77)		1.33 (0.97-1.84)		1.29 (0.94-1.78)		1.28 (0.91-1.79)	
Q4	102/1139	8605	1.43 (1.05-1.95)		1.50 (1.09-2.05)		1.43 (1.05-1.96)		1.39 (1.00-1.95)	
Q5 (Lowest)	121/1306	8531	1.49 (1.10-2.01)		1.62 (1.19-2.21)		1.56 (1.14-2.13)		1.50 (1.05-2.13)	
Index of multiple deprivation^f^										
Q1 (Least deprived)	86/1291	10 235	1 [Reference]	.04	1 [Reference]	.02	1 [Reference]	.04	1 [Reference]	.35
Q2	116/1221	9734	1.44 (1.08-1.90)		1.45 (1.09-1.92)		1.47 (1.11-1.95)		1.41 (1.06-1.87)	
Q3	97/1224	9177	1.38 (1.00-1.79)		1.36 (1.02-1.82)		1.35 (1.01-1.81)		1.27 (0.94-1.72)	
Q4	90/1109	7827	1.39 (1.03-1.87)		1.42 (1.05-1.91)		1.37 (1.02-1.85)		1.25 (0.91-1.73)	
Q5 (Most deprived)	74/913	6246	1.45 (1.06-1.99)		1.51 (1.10-2.07)		1.47 (1.07-2.10)		1.28 (0.90-1.82)	
**Age Cohort I (N = 1808; 10 484 person-years)**										
Education										
University degree	37/178	1446	1 [Reference]	.70	1	.66	1 [Reference]	.64	1 [Reference]	.30
A level	48/325	2388	0.74 (0.48-1.13)		0.72 (0.46-1.11)		0.74 (0.48-1.14)		0.70 (0.45-1.08)	
<A-level	10/82	494	0.85 (0.43-1.70)		0.89 (0.45-1.77)		0.87 (0.43-1.74		0.78 (0.39-1.55	
No qualification	144/984	6156	0.83 (0.57-1.20)		0.81 (0.55-1.18)		0.81 (0.56-1.19)		0.72 (0.49-1.07)	
Wealth^e^										
Q1 (Highest)	33/213	1630	1 [Reference]	.23	1 [Reference]	.18	1 [Reference]	.30	1 [Reference]	.21
Q2	36/230	1683	1.14 (0.71-1.82)		1.15 (0.72-1.84)		1.18 (0.74-1.90)		1.19 (0.74-1.90)	
Q3	49/289	2056	1.21 (0.77-1.89)		1.22 (0.78-1.91)		1.25 (0.78-1.98)		1.25 (0.78-1.98)	
Q4	44/322	2057	1.12 (0.71-1.76)		1.13 (0.72-1.78)		1.12 (0.69-1.81)		1.12 (0.69-1.81)	
Q5 (Lowest)	77/515	3058	1.32 (0.87-1.98)		1.37 (0.90-2.07)		1.35 (0.85-2.14)		1.35 (0.85-2.14)	
Index of multiple deprivation^f^										
Q1 (Least deprived)	49/333	2452	1 [Reference]	.55	1 [Reference]	.55	1 [Reference]	.60	1 [Reference]	.94
Q2	57/325	2285	1.30 (0.88-1.91)		1.29 (0.89-1.91)		1.31 (0.89-1.93)		1.28 (0.86-1.90)	
Q3	54/337	2197	1.34 (0.91-1.97)		1.34 (0.91-1.97)		1.34 (0.91-1.98)		1.30 (0.86-1.96)	
Q4	43/314	1964	1.15 (0.76-1.73)		1.15 (0.76-1.73)		1.13 (0.75-1.70)		1.07 (0.68-1.69)	
Q5 (Most deprived)	36/260	1586	1.21 (0.78-1.86)		1.21 (0.78-1.86)		1.20 (0.78-1.85)		1.11 (0.68-1.81)	
**Age Cohort II (N = 4412; 32 735 person-years)**										
Education										
University degree	36/798	6527	1 [Reference]	.03	1 [Reference]	.02	1 [Reference]	.04	1 [Reference]	.002
A level	55/1119	9206	1.08 (0.71-1.65)		1.12 (0.73-1.74)		1.12 (0.73-1.73)		1.02 (0.65-1.59)	
<A-level	10/234	1776	0.87 (0.43-1.77)		0.83 (0.41-1.68		0.77 (0.37-1.59		0.68 (0.33-1.41	
No qualification	123/2037	15 226	1.43 (1.01-2.04)		1.49 (1.01-2.19)		1.43 (0.97-2.11)		1.21 (0.81-1.79)	
Wealth^e^										
Q1 (Highest)	34/848	7178	1 [Reference]	.01	1 [Reference]	.001	1 [Reference]	.05	1 [Reference]	.05
Q2	46/866	6921	1.42 (0.90-2.22)		1.47 (0.93-2.31)		1.44 (0.92-2.27)		1.43 (0.89-2.29)	
Q3	42/865	6613	1.37 (0.86-2.17)		1.46 (0.92-2.31)		1.39 (0.88-2.21)		1.34 (0.81-2.19)	
Q4	58/817	6548	1.81 (1.18-2.77)		1.96 (1.28-3.01)		1.83 (1.19-2.82)		1.65 (1.02-2.70)	
Q5 (Lowest)	44/791	5475	1.73 (1.10-2.72)		2.02 (1.25-3.25)		1.82 (1.12-2.96)		1.68 (1.05-2.86)	
Index of multiple deprivation^f^										
Q1 (Least deprived)	37/958	7781	1 [Reference]	.01	1 [Reference]	.005	1 [Reference]	.01	1 [Reference]	.18
Q2	59/896	7448	1.66 (1.10-2.51)		1.67 (1.10-2.53)		1.73 (1.14-2.63)		1.62 (1.06-2.46)	
Q3	43/887	6978	1.37 (0.88-2.12)		1.39 (0.89-2.16)		1.39 (0.89-2.18)		1.27 (0.81-1.98)	
Q4	47/795	5860	1.77 (1.15-2.73)		1.82 (1.18-2.82)		1.80 (1.16-2.79)		1.55 (0.98-2.45)	
Q5 (Most deprived)	38/652	4658	1.87 (1.18-2.97)		1.98 (1.25-3.15)		1.89 (1.19-2.99)		1.50 (0.91-2.49)	

Abbreviations: Q, quintile; SES, socioeconomic indicators.

^a^ Model 1 used SES indicators analyzed individually, unadjusted.

^b^ Model 2 used 1 SES indicator at the time, adjusted for sex and marital status.

^c^ Model 3 used 1 SES indicator at the time, adjusted for sex, marital status, stroke, hypertension, diabetes, and cardiovascular disease.

^d^ Model 4 with all 3 SES indicators entered simultaneously, adjusted for sex, marital status, stroke, hypertension, diabetes, and cardiovascular disease.

^e^ In wealth rankings, Q1 indicates highest wealth category; Q2, the second highest; Q3, the third highest; Q4, the fourth highest; and Q5, the lowest.

^f^ In the index of multiple deprivation, Q1 indicates least deprived; Q2, the second least deprived; Q3, the third least deprived; Q4, the fourth least deprived; and Q5, most deprived.

Area**-**based characteristics measured with IMD were also associated with dementia incidence. In contrast with individuals in the least-deprived areas (IMD Q1), the remaining 4 quintiles showed an increase in the hazard risk of developing dementia in model 1 (Q2: HR, 1.44; 95% CI, 1.08-1.90; to Q5: HR, 1.45; 95% CI, 1.06-1.99; *P *for trend = .04). However, only the association with the second-highest quintile (Q2: HR, 1.41; 95% CI, 1.06-1.87) maintained statistical significance in the fully adjusted model, independent of the other individual markers of SES.

Results from the first sensitivity analysis showed that those in the lowest 4 quintiles of IMD combined had increased risks of developing dementia (HR, 1.32; 95% CI, 1.03-1.69; model 4) compared with those living in the least deprived area (eTable 2 in the Supplement).

### Individual and Area-Based Socioeconomic Markers Within Age Cohorts

An investigation of age cohort showed that education was significantly associated with dementia for participants born between 1926 and 1943 (age cohort II), but not for those born earlier in the century (age cohort I). In age cohort II, there was a greater hazard risk of dementia for those with no education than those educated at university levels (HR, 1.43; 95% CI, 1.01-2.04; model 1). However, this association was no longer significant once health conditions had been entered, per model 3.

Wealth also seemed to have a stronger association with dementia incidence within age cohort II, although this was not statistically significant. The association of IMD with subsequent dementia was comparable in age cohort II and the full sample, while differences between IMD quintiles were not present for age cohort I in models 1, 2, and 3, before adjusting for other SES markers.

Our additional analyses considering the competing risk of death showed a similar pattern of decline in dementia incidence over time (eFigure 2 in the Supplement) and a stronger association between dementia incidence and all the SES markers including education, but with no age-cohort effects (eTable 3 in the Supplement).

## Discussion

In a representative sample of the English population aged 65 years and older, we found a positive association between lower wealth and dementia incidence that was independent of education, area-level deprivation, and covariates. This suggests a higher risk for individuals with fewer financial resources. The association was more consistent for participants born after 1926 compared with those born earlier in the 20th century. Additionally, there was evidence for reduced incidence among participants born more recently. However, the 2 age cohorts overlap only for the group aged 75 to 79 years. Differences between age cohorts in the incidence of early-onset vs later-onset dementias may also be present.

There are several possible explanations for the strong association of wealth with subsequent health outcomes. Wealth is an indicator of socioeconomic resources, and it could represent a gateway to more mentally stimulating environments independent of the level of educational attainment. Previous ELSA findings have shown that increased wealth facilitates greater digital literacy, which is in turn associated with a reduced risk of dementia.^27^ Furthermore, increased financial status could provide broader access to cultural resources and behaviors (eg, reading, theaters, social clubs) or increased social networks, which could ultimately contribute to higher cognitive reserve.^7,28^

The integrated psychosocial resource model proposed by Matthews and Gallo^29^ argues for the accumulation of psychosocial and physical protective factors. However, in our analyses, the relationship between wealth and dementia remained statistically significant even after controlling for health**-**related conditions associated with dementia.

There is also evidence that persistent SES disadvantage is associated with impaired physiological functioning,^30^ increased risk of depression,^31^ vascular disease, and stroke.^32^ Other factors, such as reduced exercise, poor diet,^33^ and inflammatory vascular risk factors,^34^ may also play a part in the association between low SES (as defined by wealth) and increased dementia risk. Our data showed a differential SES distribution for the health conditions modeled as covariates in these analyses, except for stroke, which showed no clear SES gradient. Further work on the ELSA data could explore these mechanisms in more detail to be able to disentangle the mediating role of psychological, cardiovascular, and metabolic functions on the association between SES markers and dementia.

The lack of a contextual, area-based SES effect on dementia incidence is also notable. Previous ELSA findings have documented a link between neighborhood deprivation and cognitive functioning, independent of individual markers of SES, showing that individuals living in the most deprived area of England had significantly lower cognitive scores compared with those living in the most affluent regions.^13^ Our study found an inconsistent association between the area deprivation (IMD) and dementia incidence, with higher rates for individuals in the second quintile of IMD compared with the top quintile (who were least deprived). The reasons for this are not clear. Associations were observed for the lower IMD quintiles in first stages of covariates adjustment, but these were no longer significant when individual**-**level SES indicators were considered. This suggests that much of the effect of area deprivation is explained by the individual characteristics of the people living in those areas, rather than the features of the areas themselves.

In this cohort, education was not a robust predictor of dementia incidence. Given that this association was no longer significant after age and sex were taken into account, it is possible that this might be a specific cohort effect in the English population born and educated in the period surrounding the World War II. Support for this speculation comes from an extensive population cohort collaboration (the Epidemiological Clinicopathological Studies in Europe), which showed no apparent protective effect of education on the clinical presentation of dementia (eg, accumulation of pathology, pathological severity, and level of compensatory mechanisms for cognitive impairment).^35^ Their findings showed that individuals with higher education had heavier brains, suggesting greater cognitive reserve, but they were not necessarily able to compensate for the accumulation of vascular and neurodegenerative pathologies. However, the role of education might be sensitive to sociocultural context. Similar to our findings, other investigations from the Rotterdam Study,^36^ the Rochester Epidemiology Project,^37^ and the Baltimore Longitudinal Study of Aging^38^ reported a lack of association between dementia incidence and education.

In contrast, findings from the Health and Retirement Study^39^ indicated that higher education was associated with a lower risk of dementia prevalence between 2000 and 2012, and in the Kungsholmen study,^40^ education remained significantly associated with dementia following adjustment for occupational class. Moreover, in the Canadian Study of Health and Aging,^41^ fewer years of education were associated with an increased risk of late**-**onset Alzheimer disease incidence, while subsequent results from a 10-year follow-up (1991-2001) within the same study showed that high complexity of work with people or things was associated with a reduced risk of most dementia types (Alzheimer and vascular dementia).^42^ These findings indicate a protective effect of the occupational demands on the brain achieved through a lifetime occupational exposure. It is therefore possible that individuals born before the World War II may not necessarily have been able to access higher education (because of military service, financial restrictions, and limited university place availability) but may have gained access to intellectually challenging jobs and growth opportunities after the war.

### Strengths

To our knowledge, this is the first longitudinal study to examine multiple facets of SES characteristics at individual and group levels simultaneously in association with dementia incidence within an age-cohort context. Through the extensive monitoring of biennial interviews and a long-term follow-up, we were able to use an integrative approach to study the association between various socioeconomic factors and dementia incidence. Furthermore, we benefited from a more detailed assessment of wealth than what is available in most studies to date, because this measure was computed on the basis of accurate information on multiple individual components rather than broad categorization of assets.

### Limitations

This study also has limitations. Given that the ascertainment of dementia diagnosis is still challenging in the UK health services and elsewhere, it is likely that the presented dementia IRs are underestimated. Other common issues such as nonresponse and subsequent attrition are familiar to most longitudinal surveys.^43^ Moreover, because of a relatively small sample of dementia cases, we did not explore the IRs of dementia by specific typology (eg, Alzheimer disease, vascular, mixed). Although ELSA is a demographically representative cohort, the race/ethnicity is 97% white^16^ and we were therefore unable to investigate the effects that race/ethnicity might have on the outcome of dementia. Furthermore, we did not investigate the difference in dementia incidence by geographical regions, given the high collinearity with IMD. Lastly, as in any observational study, we cannot exclude the risk of confounding by other factors. Avenues for future exploration include examining the mediating role of cardiovascular disease, lifestyle factors, medical care and other risk factors that could influence the association between SES and dementia.

## Conclusions

In a nationally representative sample of English people 65 years and older, the hazard risk of dementia incidence was associated with socioeconomic indicators, notably wealth. Socioeconomic inequalities were more marked in individuals born in later years (from 1926 onwards) than in those born earlier (between 1900 and 1925). Public health strategies for dementia prevention should target socioeconomic gaps to reduce health disparities and protect those who are particularly disadvantaged in addition to addressing vascular risk factors such as hypertension, diabetes mellitus, smoking, and heart disease.

## Notes

Supplement. **eAppendix.** Supplementary materials description. **eFigure 1.** Flowchart of the individuals included in the competing risk hazard model analyses. **eFigure 2.** Stacked cumulative incidence plots for dementia and death as competing risks within each age cohort. **eTable 1.** Dementia incidence rates per 1,000 person-years and 95% confidence intervals (CI), according to baseline characteristics in the overall ELSA sample (N=6220; 463 dementia cases). **eTable 2.** Hazard Ratios (HR) and 95% confidence intervals (CI) from univariate and multivariate Cox regression models by age. **eTable 3.** Hazard Ratios (HR) and 95% confidence intervals (CI) from univariate and multivariate Fine and Gray Subdistribution Hazards model adjusting for the competing risk of death.
