# Supplementary material for: Individual and Area-Based Socioeconomic Factors Associated With Dementia Incidence in England: Evidence From a 12-Year Follow-up in the English Longitudinal Study of Ageing
Source: JAMA Psychiatry. 2018 May 16;75(7):723–32. doi: 10.1001/jamapsychiatry.2018.1012 (PMC6145673; doi:10.1001/jamapsychiatry.2018.1012)
Supplement: Supplement. — eAppendix. Supplementary materials description. eFigure 1. Flowchart of the individuals included in the competing risk hazard model analyses. eFigure 2. Stacked cumulative incidence plots for dementia and death as competing risks within each age cohort. eTable 1. Dementia incidence rates per 1,000 person-years and 95% confidence intervals (CI), according to baseline characteristics in the overall ELSA sample (N=6220; 463 dementia cases). eTable 2. Hazard Ratios (HR) and 95% confidence intervals (CI) from univariate and multivariate Cox regression models by age. eTable 3. Hazard Ratios (HR) and 95% confidence intervals (CI) from univariate and multivariate Fine and Gray Subdistribution Hazards model adjusting for the competing risk of death. [file jamapsychiatry-e181012-s001.pdf]

Supplementary Online Content

Cadar D, Lassale C, Davies H, Llewellyn DJ, Batty GD, Steptoe A. Individual and area-based socioeconomic factors associated with dementia incidence in England: evidence from a 12-year follow-up in the English Longitudinal Study of Ageing. *JAMA Psychiatry*. Published online May 16, 2018. doi:10.1001/jamapsychiatry.2018.1012

eAppendix. Supplementary materials description.

eFigure 1. Flowchart of the individuals included in the competing risk hazard model analyses

eFigure 2. Stacked cumulative incidence plots for dementia and death as competing risks within each age cohort

eTable 1. Dementia incidence rates per 1000 person-years and 95% confidence intervals (CI), according to baseline characteristics in the overall ELSA sample (N=6220; 463 dementia cases)

eTable 2. Hazard Ratios (HR) and 95% confidence intervals (CI) from univariate and multivariate Cox regression models by age

eTable 3. Hazard Ratios (HR) and 95% confidence intervals (CI) from univariate and multivariate Fine and Gray Subdistribution Hazards model adjusting for the competing risk of death

This supplementary material has been provided by the authors to give readers additional information about their work.

© 2018 Cadar D, et al. *JAMA Psychiatry*.

eAppendix. Supplementary materials description.

There were significant differences between individuals with cardiovascular disease by levels of education, χ^2 (3), 7.60, P≤.05, wealth, χ^2 (4), 29.00, P≤.001 and IMD, χ^2 (4), 26.30, P≤.001. Significant differences were also observed between individuals with diabetes by levels of education, χ^2 (3), 12.22, P≤.05, wealth, χ^2 (4), 16.74, P≤.002 and IMD, χ^2 (4), 38.62, P≤.001 and those with hypertension by levels of education, χ^2 (3), 16.74, P≤.001, wealth, χ^2 (4), 22.35, P≤.001 and IMD, χ^2 (4), 14.67, P≤.005.

Table 1 presents dementia rates by gender and Table 2 presents the results of the sensitivity analysis conducted with the index of multiple deprivation (IMD) categories regrouped into a binary variable. Supplementary analyses were conducted using a modification of the Fine and Gray, Subdistribution Hazards model [31], described by Chang and colleagues [32].

In the first stage of these analyses, we constructed a propensity model to estimate the probability of having dementia at the last observed follow-up for each participant, using a logistic regression model in which we included education, wealth, IMD, sex, marital status, stroke, hypertension, diabetes and CVD. Age was not included because we investigated age-cohort effects in these analyses. In this propensity model, those who developed dementia were considered as "events", while participants who remained alive and not classified with dementia until the end of the study, were considered "non-events". Each individual (including those who died without developing dementia) was assigned a corresponding propensity score by calculating the estimated probability.

In the second stage, the propensity scores for those who died without developing dementia were used to reclassify them into the main event (dementia or death with a high probability of having dementia) or competing risk event (death with a low probability of having dementia). The threshold used for the reclassification of those who died into high versus the low probability of having had dementia was the median propensity score among those who developed dementia (median=0.11). Individuals who died were reclassified as having had dementia if their propensity score was above the threshold, or as competing event (death with a low probability of dementia) if their propensity score was below the

© 2018 Cadar D, et al. *JAMA Psychiatry.*

threshold (see Figure 1 for the flowchart). As a result, in this overall sample of 6220 participants, the number of people classified with dementia or having died with a high probability of dementia was N = 1607 (463 original dementia cases and 1144 individuals who died with a high probability of dementia before they died), while the number of people who died with a low probability of dementia was N=827. The cumulative incidence of dementia and death as competing risks within each age cohort are presented in Figure 2. The results of the association between all SES indicators and dementia incidence, while accounting for the competing risk of death are presented in Table 3.

© 2018 Cadar D, et al. *JAMA Psychiatry*.

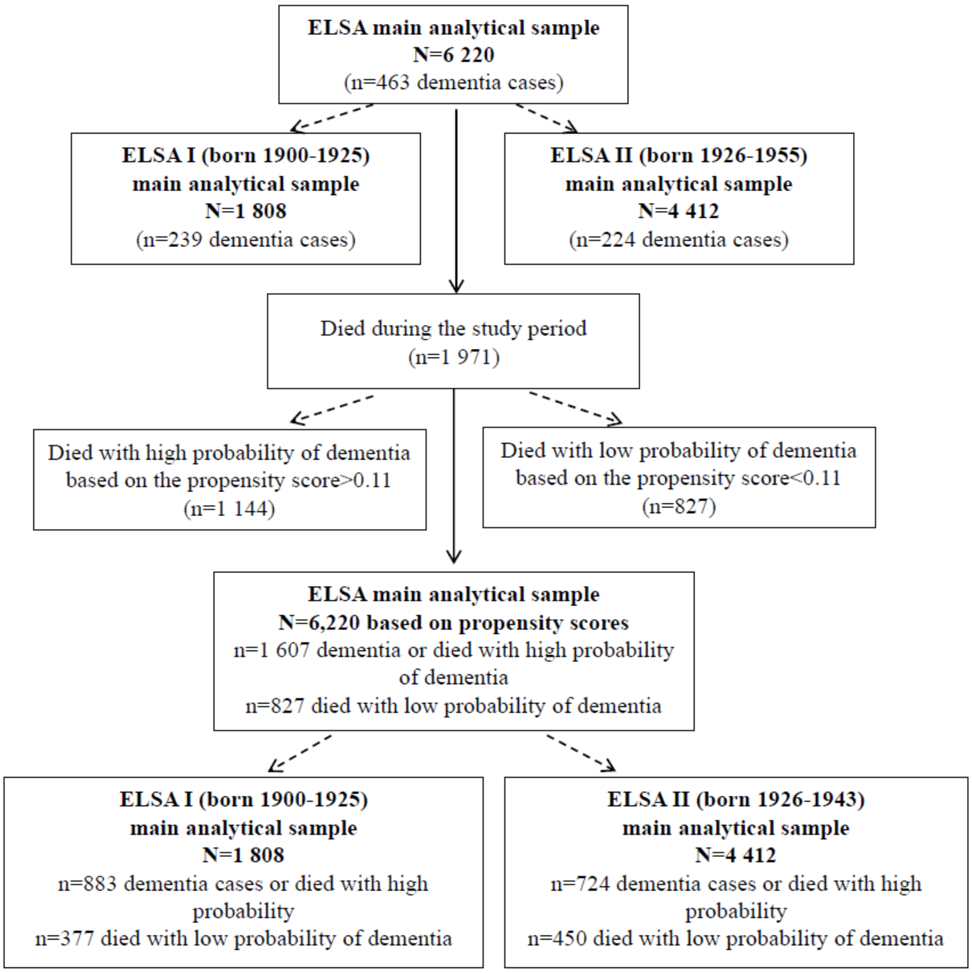

Figure 1. Flowchart of the individuals included in the competing risk hazard model analyses

© 2018 Cadar D, et al. *JAMA Psychiatry.*

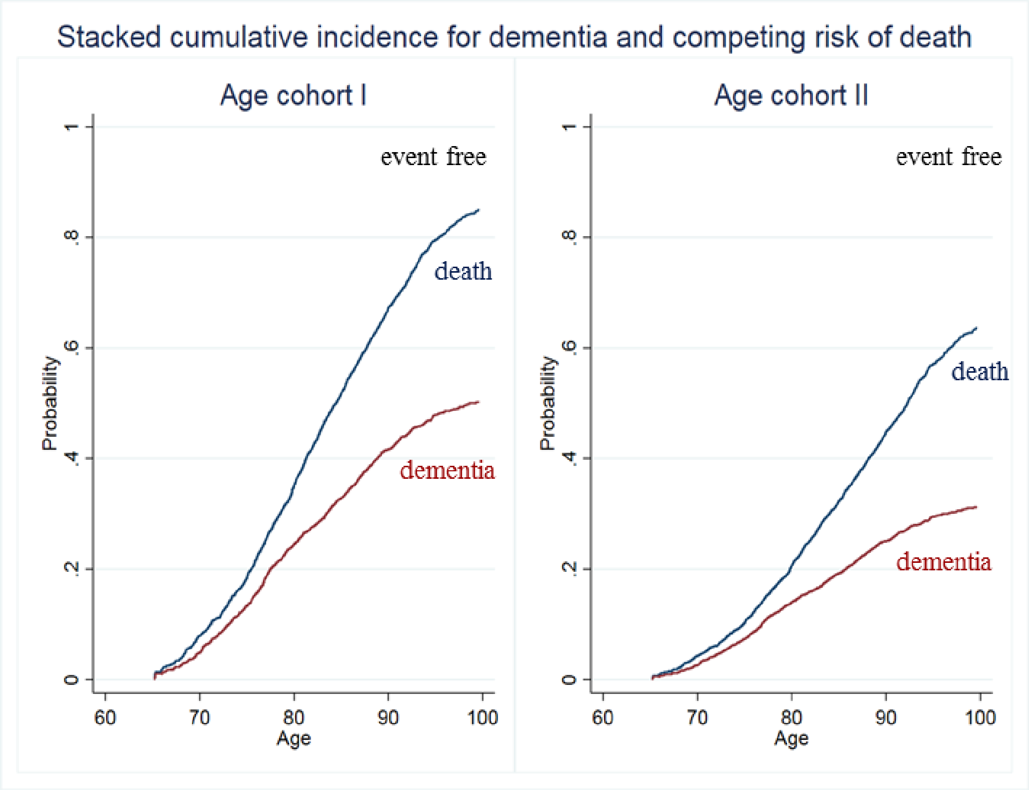

Figure 2. Stacked cumulative incidence plots for dementia and death as competing risks within each age cohort

© 2018 Cadar D, et al. *JAMA Psychiatry*.

Table 1. Dementia incidence rates per 1 000 person-years and 95% confidence intervals (CI), according to baseline characteristics in the overall ELSA sample (N=6 220; 463 dementia cases)

	Overall (N=6 220)			Men (N=2 810)			Women (N=3 410)		
	N (cases/ censored)	Rate	95% CI	N (cases/ censored)	Rate	95% CI	N (cases/ censored)	Rate	95% CI
Age continuous	463/5757	11.32	10.34-12.41	187/2623	10.27	8.92-11.89	276/3134	12.09	10.76-13.63
Age-groups									
65-69	71/2008	4.38	3.49-5.57	36/948	4.86	3.54-6.85	35/1060	3.97	2.88-5.63
70-74	105/1705	8.30	6.88-10.08	48/797	8.50	6.46-11.41	57/908	8.13	6.33-10.63
75-79	127/963	17.14	14.49-20.41	50/459	15.53	11.88-20.67	77/534	18.24	14.74-22.83
80+	160/1051	24.69	21.20-28.91	53/419	22.85	17.64-30.09	107/632	25.62	21.26-31.13
Marital status									
Married	254/3428	9.94	8.81-11.26	139/1908	10.06	8.55-11.92	115/1520	9.81	8.21-11.81
Single/divorced	209/2329	13.35	11.68-15.33	48/715	10.92	8.31-14.63	161/1614	14.20	12.19-16.64
Education									
Higher	73/976	9.85	7.86-12.50	30/563	6.58	4.64-9.63	43/413	13.88	10.40-18.94
A level	103/1444	9.17	7.60-11.18	45/598	10.21	7.69-13.85	58/846	8.51	6.63-11.10
Below A-level	20/316	9.71	6.31-15.70	17/272	9.50	5.98-15.99	3/44	10.85	3.08-32.32
No qualification	267/3021	13.08	11.62-14.77	95/1190	12.44	10.23-15.27	172/1831	13.43	11.58-15.65
Wealth (quintiles)									
Q1 (highest)	67/1062	7.92	6.26-10.16	35/546	8.10	5.87-11.49	32/515	7.74	5.52-11.18
Q2	82/1096	10.11	8.16-12.67	31/522	8.01	5.68-11.65	51/574	11.80	9.01-15.75
Q3	91/1154	11.03	9.02-13.64	41/511	11.40	8.43-15.76	50/643	10.77	8.22-14.36
Q4th	102/1139	12.54	10.38-15.28	38/513	11.16	8.22-15.51	64/626	13.50	10.65-17.37
Q5 (lowest)	121/1306	15.05	12.62-18.10	42/529	13.51	10.03-18.63	79/776	15.91	12.81-19.99
IMD (quintiles)									
Q1 (least deprived)	86/1291	8.62	7.48-11.24	42/574	9.56	7.13-11.09	44/716	7.90	5.93-10.75
Q2	116/1221	12.47	9.50-14.06	52/562	12.08	9.26-16.07	64/659	12.77	10.04-16.47
Q3	97/1224	11.56	10.20-15.10	35/570	9.33	6.76-13.25	62/654	13.17	10.32-17.07
Q4	90/1109	11.99	9.21-13.88	36/501	11.19	8.19-15.69	54/608	12.58	9.73-16.56
Q5 (most deprived)	74/913	12.64	10.70-16.86	22/416	8.77	5.84-13.74	52/497	15.29	11.70-20.33
Stroke									

© 2018 Cadar D, et al. *JAMA Psychiatry*.

No	407/5170	10.87	9.87-11.99	161/2333	9.69	8.33-11.35	246/2837	11.73	10.37-13.32
Yes	56/587	16.47	12.78-21.56	26/290	16.38	11.33-24.52	30/297	16.55	11.71-24.11
Hypertension									
No	240/3239	11.27	9.93-12.85	97/1564	9.38	7.69-11.56	143/1675	12.81	10.88-15.19
Yes	223/2518	12.44	10.86-14.27	90/1059	12.63	10.26-15.73	133/1459	12.32	10.39-14.72
Diabetes									
No	416/5215	11.12	10.11-12.26	164/2330	10.04	8.64-11.73	252/2885	11.90	10.53-13.49
Yes	47/542	13.52	10.28-18.13	23/293	12.42	8.38-19.18	24/249	14.66	10.05-22.23
CVD									
No	373/5081	10.17	9.15-11.34	142/2267	9.01	7.59-10.75	231/2814	10.97	9.59-12.61
Yes	90/676	16.21	13.67-19.36	45/356	14.76	11.51-19.23	45/320	17.62	13.97-22.50

© 2018 Cadar D, et al. *JAMA Psychiatry*.

Table 2. Hazard Ratios (HR) and 95% confidence intervals (CI) from univariate and multivariate Cox regression models by age

	**ELSA Overall (N=6 220) 43 219 person-years**				**Age cohort I (N=1 808) 10 484 person-years**				**Age cohort II (N=4 412) 32 735 person-years**			
	N (cases/ censored)	Person years	HR	95% CI	N (cases/ censored)	Person years	HR	95% CI	N (cases/ censored)	Person years	HR	95% CI
Model 1 with SES indicators analysed individually, unadjusted												
IMD												
Q1 (least deprived)	86/1291	10235	1	-	49/333	2452	1	-	37/958	7,781	1	-
Q2-Q5	377/4466	32984	1.40	1.11-1.77	139/908	8032	1.32	0.87-2.01	187/3229	24,954	1.64	1.15-2.34
Model 2 with one SES indicator at the time, adjusted for sex and marital status												
IMD												
Q1 (least deprived)	86/1291	10235	1	-	100/661	2452	1	-	37/958	7,781	1	-
Q2-Q5	377/4466	32984	1.43	1.13-1.81	139/908	8032	1.25	0.97-1.63	187/3229	24,954	1.68	1.17-2.40
Model 3 with one SES indicator at the time, adjusted for sex, marital status, stroke, hypertension, diabetes and CVD												
IMD												
Q1 (least deprived)	86/1291	10235	1	-	100/661	2452	1	-	37/958		1	-
Q2-Q5	377/4466	32984	1.41	1.12-1.79	139/908	8032	1.24	0.95-1.61	187/3229		1.68	1.17-2.41
Model 4 with all 3 SES indicators entered simultaneously, adjusted for sex, marital status, stroke, hypertension, diabetes and CVD												
IMD												
Q1 (least deprived)	86/1291	10235	1	-	100/661	2452	1	-	37/958	7,781	1	-
Q2-Q5	377/4466	32984	1.32	1.03-1.69	139/908	8032	1.23	0.92-1.63	187/3229	24,954	1.48	1.03-2.14

© 2018 Cadar D, et al. *JAMA Psychiatry*.

Table 3. Hazard Ratios (HR) and 95% confidence intervals (CI) from univariate and multivariate Fine and Gray Subdistribution Hazards model adjusting for the competing risk of death

	**ELSA Overall (N=6 220) 43 219 person-years**			**Age cohort I (N=1 808) 10 484 person-years**			**Age cohort II (N=4 412) 32 735 person-years**		
	N (cases/death/ censored)	HR	95% CI	N (cases/death/ censored)	HR	95% CI	N (cases/death/ censored)	HR	95% CI
Model 1 with SES indicators analysed individually, unadjusted									
Education									
University degree	144/182/723	1	-	72/71/72	1	-	72/111/651	1	-
A level	239/260/1,048	1.09	0.89-1.36	115/131/127	0.89	0.66-1.21	124/129/921	1.25	0.93-1.67
Below A-level	95/49/192	1.14	1.64-2.80	46/19/27	2.01	1.36-2.98	49/30/165	2.27	1.56-3.27
No qualification	1129/336/1823	2.77	2.14-3.31	650/156/322	2.42	1.87-3.12	479/180/1501	2.83	2.21-3.63
			p trend≤.001			*p trend≤.001*			*p trend≤.001*
Wealth									
Q1 (highest)	113/208/808	1	-	60/97/89	1	-	53/111/718	1	-
Q2	173/214/791	1.59	1.25-2.03	88/95/83	1.59	1.13-2.23	85/119/708	1.37	0.97-1.94
Q3	292/149/804	2.73	2.19-3.40	160/62/116	2.69	1.99-3.66	132/87/688	1.80	1.29-2.49
Q4	403/137/701	3.78	3.06-4.67	202/65/99	3.35	2.49-4.52	201/72/602	2.29	1.67-3.15
Q5 (lowest)	625/119/683	5.46	4.47-6.71	372/58/162	4.65	3.51-6.15	253/61/522	2.69	1.94-3.73
			p trend≤.001			*p trend≤.001*			*p trend≤.001*
IMD									
Q1 (least deprived)	215/254/908	1	-	122/122/138	1	-	93/132/770	1	-
Q2	279/209/849	1.44	1.19-1.72	159/111/112	1.47	1.15-1.87	120/98/737	1.39	1.06-1.82
Q3	346/178/797	1.96	1.64-2.33	211/65/115	2.20	1.74-2.78	135/113/682	1.67	1.28-2.18
Q4	390/120/689	2.65	2.24-3.14	208/52/97	2.56	2.04-3.22	182/68/592	2.69	2.09-3.45
Q5 (most deprived)	376/66/544	3.35	2.82-3.97	183/27/86	2.98	2.37-3.76	193/39/458	3.62	2.82-4.65
			p trend≤.001			*p trend≤.001*			*p trend≤001*
Model 2 with one SES indicator at the time, adjusted for sex and marital status									
Education									
University degree	144/182/723	1	-	72/71/72	1	-	72/111/651	1	-

© 2018 Cadar D, et al. *JAMA Psychiatry*.

A level	239/260/1048	1.05	0.85-1.30	115/131/127	0.80	0.58-1.09	124/129/921	1.28	0.96-1.73
Below A-level	95/49/192	2.31	1.78-2.98	46/19/27	2.30	1.60-3.32	49/30/165	2.19	1.52-3.16
No qualification	1129/336/1823	2.54	2.12-3.04	650/156/322	2.15	1.66-2.79	479/180/1501	2.75	2.13-3.54
			p trend≤.001			*p trend≤.001*			*p trend≤.001*
Wealth									
Q1 (highest)	113/208/808	1	-	60/97/89	1	-	53/111/718	1	-
Q2	173/214/791	1.46	1.14-1.86	88/95/83	1.41	1.00-1.99	85/119/708	1.54	1.09-2.18
Q3	292/149/804	2.49	2.00-3.10	160/62/116	2.59	1.90-3.52	132/87/688	2.42	1.75-3.33
Q4th	403/137/701	3.28	2.65-4.06	202/65/99	3.11	2.31-4.21	201/72/602	3.50	2.58-4.74
Q5 (lowest)	625/119/683	4.05	3.29-4.97	372/58/162	3.63	3.73-4.83	253/61/522	4.74	3.50-6.43
			p trend≤.001			*p trend≤.001*			*p trend≤.001*
IMD									
Q1 (least deprived)	215/254/908	1	-	122/122/138	1	-	93/132/770	1	-
Q2	279/209/849	1.40	1.17-1.68	159/111/112	1.45	1.14-1.85	120/98/737	1.33	1.01-1.74
Q3	346/178/797	1.85	1.55-2.20	211/65/115	2.06	1.64-2.59	135/113/682	1.60	1.22-2.08
Q4	390/120/689	2.43	2.06-2.87	208/52/97	2.43	1.95-3.04	182/68/592	2.39	1.87-3.07
Q5 (most deprived)	376/66/544	2.77	2.34-3.28	183/27/86	2.56	2.04-3.21	193/39/458	2.99	2.32-3.85
			p trend≤.001			*p trend≤.001*			*p trend≤.001*
Model 3 with one SES indicator at the time, adjusted for sex, marital status, stroke, hypertension, diabetes and CVD									
Education									
University degree	144/182/723	1	-	72/71/72	1	-	72/111/651	1	-
A level	239/260/1048	1.04	0.84-1.28	115/131/127	0.81	0.60-1.10	124/129/921	1.24	0.93-1.66
Below A-level	95/49/192	2.14	1.65-2.79	46/19/27	2.20	1.52-3.20	49/30/165	1.99	1.37-2.89
No qualification	1129/336/1823	2.42	2.02-2.90	650/156/322	2.13	1.64-2.75	479/180/1501	2.54	1.97-3.27
			p trend≤.001			*p trend≤.001*			*p trend≤.001*
Wealth									
Q1 (highest)	113/208/808	1	-	60/97/89	1	-	53/111/718	1	-
Q2	173/214/791	1.44	1.13-1.82	88/95/83	1.40	1.00-1.96	85/119/708	1.51	1.07-2.13
Q3	292/149/804	2.35	1.88-2.92	160/62/116	2.47	1.83-3.35	132/87/688	2.26	1.64-3.11
Q4th	403/137/701	3.11	2.52-3.84	202/65/99	2.97	2.21-4.01	201/72/602	3.26	2.41-4.41
Q5 (lowest)	625/119/683	3.76	3.07-4.62	372/58/162	3.52	2.66-4.66	253/61/522	4.09	3.02-5.56
			p trend≤.001			*p trend≤.001*			*p trend≤.001*

© 2018 Cadar D, et al. *JAMA Psychiatry.*

IMD									
Q1 (least deprived)	215/254/908	1	-	122/122/138	1	-	93/132/770	1	-
Q2	279/209/849	1.42	1.18-1.70	159/111/112	1.46	1.15-1.85	120/98/737	1.37	1.05-1.81
Q3	346/178/797	1.85	1.54-1.18	211/65/115	2.04	1.63-2.56	135/113/682	1.61	1.24-2.11
Q4	390/120/689	2.28	1.93-2.70	208/52/97	2.32	1.85-2.89	182/68/592	2.27	1.77-2.92
Q5 (most deprived)	376/66/544	2.63	2.22-3.11	183/27/86	2.48	1.97-3.11	193/39/458	2.78	2.16-3.60
	p trend≤.001			*p trend≤.001*			*p trend≤.001*		
Model 4 with all 3 SES indicators entered simultaneously, adjusted for sex, marital status, stroke, hypertension, diabetes and CVD									
Education									
University degree	144/182/723	1	-	72/71/72	1	-	72/111/651	1	-
A level	239/260/1048	0.88	0.71-1.09	115/131/127	0.73	0.54-0.99	124/129/921	1.00	0.74-1.35
Below A-level	95/49/192	1.51	1.15-1.99	46/19/27	1.56	1.06-2.30	49/30/165	1.38	0.94-2.05
No qualification	1129/336/1823	1.65	1.37-1.99	650/156/322	1.53	1.17-1.99	479/180/1501	1.65	1.26-2.16
	p trend≤.001			*p trend≤.001*			*p trend≤.001*		
Wealth									
Q1 (highest)	113/208/808	1	-	60/97/89	1	-	53/111/718	1	-
Q2	173/214/791	1.33	1.06-1.68	88/95/83	1.31	0.95-1.81	85/119/708	1.39	0.98-1.96
Q3	292/149/804	1.87	1.50-2.34	160/62/116	1.93	1.43-2.62	132/87/688	1.83	1.31-2.54
Q4th	403/137/701	2.24	1.81-2.77	202/65/99	2.15	1.60-2.89	201/72/602	2.33	1.69-3.20
Q5 (lowest)	625/119/683	2.52	2.03-3.12	372/58/162	2.35	1.76-3.13	253/61/522	2.74	1.97-3.81
	p trend≤.001			*p trend≤.001*			*p trend≤.001*		
IMD									
Q1 (least deprived)	215/254/908	1	-	122/122/138	1	-	92/133/770	1	-
Q2	279/209/849	1.23	1.03-1.46	159/111/112	1.24	0.98-1.57	102/116/737	1.22	0.93-1.61
Q3	346/178/797	1.40	1.17-1.66	211/65/115	1.51	1.20-1.89	99/149/682	1.30	1.00-1.97
Q4	390/120/689	1.53	1.29-1.82	208/52/97	1.55	1.23-1.96	124/126/592	1.56	1.20-2.02
Q5 (most deprived)	376/66/544	1.54	1.29-1.85	183/27/86	1.49	1.17-1.91	128/104/458	1.63	1.23-2.14
	p trend≤.001			*p trend≤.001*			*p trend≤ .001*		

© 2018 Cadar D, et al. *JAMA Psychiatry*.
